# Clinical and Public Health Challenge of Handling Synthetic Cathinone and Cannabinoid Abuse in Pediatric Care: A Narrative Review

**DOI:** 10.3390/pediatric17010019

**Published:** 2025-02-08

**Authors:** Valerio Ricci, Giuseppe Maina

**Affiliations:** 1 Psychiatry Department, San Luigi Gonzaga Hospital, University of Turin, Regione Gonzole, 10, 10043 Orbassano, Italy; giuseppe.maina@unito.it; 2 Department of Neurosciences “Rita Levi Montalcini”, University of Turin, Via Cherasco n. 15, 10126 Torino, Italy

**Keywords:** synthetic cannabinoids, synthetic cathinones, psychosis, k2, spice, designer drugs in children, seizures, pediatric toxicology

## Abstract

Background: Synthetic cathinones and cannabinoids have emerged as significant public health concerns, particularly in pediatric populations. Marketed under deceptive names such as “bath salts” and “K2/Spice”, these substances pose unique challenges due to their accessibility, potency, and unpredictable effects. This narrative review synthesizes evidence on the toxicological effects of synthetic cathinones and cannabinoids in pediatric patients, emphasizing clinical presentations, management challenges, and public health implications. Methods: A structured narrative review was conducted using PubMed and Scopus databases to identify peer-reviewed studies published between January 2010 and September 2024. The selected articles focus on neuropsychiatric, systemic, and management outcomes associated with these substances in individuals aged 0–18 years. Results: Five studies demonstrate that synthetic cathinones frequently cause seizures, sympathomimetic toxidrome (tachycardia, hypertension), and neuropsychiatric effects like paranoia and catatonia. Seven studies show synthetic cannabinoids induce psychosis, respiratory depression requiring ventilation in 12% of cases, and cardiovascular complications like myocardial ischemia. One study highlighted severe outcomes in pediatric accidental exposures, emphasizing the unpredictable and life-threatening effects of these substances, often exacerbated by co-ingestion with alcohol or THC. Conclusions: Pediatric exposure to synthetic cathinones and cannabinoids results in severe and unpredictable toxicological effects, necessitating tailored clinical management strategies and enhanced diagnostic capabilities. Public health measures, including stringent regulatory controls, targeted education initiatives, and robust surveillance systems, are critical to mitigating these risks. A multidisciplinary approach is essential to safeguard vulnerable pediatric populations from the escalating dangers posed by synthetic drugs, and future research must address the long-term impacts and mechanisms of toxicity.

## 1. Introduction

Between 2009 and 2017, a total of 803 new psychoactive substances (NPS) were reported across 111 countries and territories [1]. Within the European Union, the number of identified NPS surpassed 670 by the end of 2017, with 632 substances recorded since 2004 [2]. The majority of these substances included synthetic cannabinoids, synthetic cathinones, phenethylamine derivatives, and synthetic opioids.

Although organizations such as the European Monitoring Centre for Drugs and Drug Addiction (EMCDDA) and the United Nations Office on Drugs and Crime (UNODC) monitor and classify NPS, the actual scope of these substances may be significantly broader than the cases formally documented by international agencies. In this context, exploring discussions within online communities of NPS enthusiasts, often referred to as “e-psychonauts” [3], can provide valuable insights into the availability, market trends, and distribution of these substances. Notably, online trends often anticipate real-world developments in the NPS market [4]. For instance, a link between NPS use and violent behavior was identified among patients admitted to acute mental health services in London, UK [5]. Similarly, the psychopathological and aggressive behaviors associated with drug use in Ibiza’s club scene [6] had been previously anticipated through studies observing evolving patterns in the “e-psychonaut” community [7]. It is important to note that the UNODC only includes an NPS in its database once the substance has been seized, chemically analyzed, and formally reported.

Synthetic cathinones and cannabinoids have emerged as a critical public health concern, particularly in the pediatric population. These substances, often marketed under deceptive names such as “bath salts”, have gained popularity due to their accessibility, affordability, and the perception of legality [8,9,10]. First gaining prominence in Europe and the United States in the early 2010s, synthetic cathinones and cannabinoids have since become widely abused, posing significant medical and societal challenges [11].

Cathinones are phenylalkylamine alkaloids, also referred to as A-ketone amphetamine analogs, naturally found in the leaves and stems of the khat plant (*Catha edulis*) [12,13]. *Catha edulis* is a slow-growing shrub native to Ethiopia, commonly cultivated in East Africa and the southwestern Arabian Peninsula. Chewing the leaves of this plant produces psychostimulant effects and has been associated with a withdrawal syndrome [14,15,16]. The synthesis of cathinone derivatives was first documented in the 1920s, with methcathinone synthesized in 1928 and mephedrone in 1929 [17,18]. Although these derivatives have been explored for medical applications, most were abandoned due to severe side effects. The exception is bupropion, a cathinone derivative approved for the treatment of depression and as an aid for smoking cessation, which remains the only medically approved cathinone in the United States and Europe [19]. Synthetic cathinones began gaining traction among recreational users sometime before 2007, with early reports of such usage appearing on Internet forums [20]. Synthetic cathinones are phenylalkylamine derivatives characterized by the presence of a ketone at the A position of the amino alkyl chain attached to the phenyl ring, earning them the designation bk-amphetamines [21]. Their stimulant effects are comparable to methylphenidate at lower doses and resemble cocaine or amphetamines at higher doses. Like amphetamines, synthetic cathinones increase synaptic catecholamine levels, including dopamine, serotonin, and norepinephrine, thereby inducing stimulant effects [22]. However, these substances are typically less potent than amphetamines due to their A-keto group, which increases polarity and reduces their ability to cross the blood-brain barrier. Although research is ongoing, the metabolism of synthetic cathinones is not yet fully understood [23]. Most appear to be metabolized in the liver and excreted in the urine [23].

Among the expanding range of synthetic cathinones, three substances stand out in medical and media reports: MDPV (3,4-methylenedioxypyrovalerone), methylone (3,4-methylenedioxy-N-methylcathinone), and mephedrone (4-methylmethcathinone). These were temporarily classified under Schedule I of the Controlled Substances Act in 2011. Mephedrone is the most commonly used synthetic cathinone in Europe, while MDPV is more prevalent in the United States [24]. The cultural practice of chewing khat leaves for stimulant effects has been documented since the 11th century, with modern use primarily in Yemen and the Middle East. In 2006, the global daily user population was estimated at 10 million [25,26,27,28]. Cathinone, a central nervous system stimulant with lower potency than amphetamines, increases heart rate and blood pressure, induces euphoria, enhances alertness, and boosts psychomotor activity [29]. However, khat use is linked to adverse effects, including hypertension, myocardial infarction, gastrointestinal issues, liver toxicity, and insomnia [30,31].

Synthetic cannabinoid (SC) or synthetic marijuana (Spice/k2) are widely accessible through online platforms, gas stations, and convenience stores [32,33,34]. Introduced in Europe in 2004 as a legal alternative to marijuana, it is commonly associated with individuals presenting psychiatric symptoms such as anxiety, agitation, and psychosis, often without a prior psychiatric history [35,36]. It consists of dried plant material laced with synthetic cannabinoid receptor agonists [37,38,39]. Several synthetic cannabinoid compounds have been identified in Spice, some of which are prohibited in the United States and Europe. As legislation evolves to ban certain substances, manufacturers adapt by incorporating chemically similar, yet unregulated, compounds [2]. Synthetic cannabinoids were initially developed in 1965 to replicate the effects of tetrahydrocannabinol (THC). Research led to three notable medications: dronabinol (Marinol), a synthetic THC for pain and antiemetic use; nabilone (Cesamet), for anorexia and nausea; and rimonabant (Acomplia), a CB1 receptor antagonist used to manage obesity but later withdrawn due to adverse effects [33]. These compounds interact with CB1 and CB2 cannabinoid receptors. CB1, predominantly found in the central nervous system (CNS), mediates cannabis’s psychotropic and physiological effects, while CB2, located in the CNS and immune cells, primarily affects immune functions [34,35]. The use of cannabinoids in pain management presents several challenges. One significant concern is the method of drug delivery. Smoking cannabis is the most straightforward method and allows for effective dose control [40,41]. However, smoking is associated with well-known adverse effects, and the THC content of cannabis can vary depending on cultivation practices and environmental factors. In Canada, where medicinal cannabis is approved for chronic pain treatment, patients are required to obtain their cannabis from centralized suppliers to ensure consistent monitoring of THC levels over time [41]. Similar regulatory measures are being considered in other parts of the world, including the United States.

Synthetic cannabinoids, such as dronabinol and nabilone, are approved for use in the United States and Canada as antiemetics during cancer chemotherapy and as adjunct therapies for neuropathic and chronic pain. However, their use remains limited, and further studies are underway to better delineate their efficacy and pharmacological profiles in different clinical contexts [42]. Reported side effects include elevated heart rate, changes in blood pressure, anxiety, psychomotor slowing, memory impairment, and psychosis. These compounds are contraindicated in specific conditions such as pregnancy, uncontrolled hypertension, active ischemic heart disease, arrhythmias, and schizophrenia. Clark et al. have outlined guidelines for the clinical use of cannabinoid-based therapies [43]. Cannabis and cannabinoids also carry a potential for dependence, which can result in compulsive use and preoccupation. When prescribing cannabinoids for chronic pain, clinicians must carefully select patients and closely monitor their use to prevent the development of dependency [40,41]. Recent research by Donoghue et al. examines the interaction between cannabis use, gender, and the age of onset of schizophrenia and schizoaffective disorders. Their findings indicate that cannabis use is linked to an earlier onset of schizophrenia and that the gender differences in age of onset are reduced in cannabis users [44].

Synthetic cannabinoids are divided into four groups by chemical structure. JWH compounds, like JWH-018, have higher receptor affinity than THC, while CP and HU compounds are even more potent, with HU compounds being up to 800 times stronger [45]. Routine drug screens often miss these substances. Users report effects similar to marijuana, such as euphoria, relaxation, and altered perception, with symptoms like red eyes, tachycardia, and dry mouth lasting up to 6 h [46,47]. Severe reactions include agitation, anxiety, seizures, psychosis, and suicidal ideation [48,49,50].

A case series found psychotic symptoms lasting up to eight days in seven patients, with three showing persistent psychosis for over five months [51,52,53,54,55]. Synthetic cannabinoids may induce acute or chronic psychosis, especially in vulnerable individuals [56].

The pediatric population is particularly vulnerable to the toxicological impacts of these substances due to their developmental susceptibility and higher likelihood of experimental use. Emergency physicians have reported cases of severe intoxication in adolescents, including psychosis, seizures, and life-threatening cardiovascular events, often necessitating intensive care [57,58]. Additionally, the absence of routine toxicological screening for these substances complicates diagnosis and delays intervention [59].

This narrative review aims to provide a comprehensive synthesis of the current understanding of synthetic cathinones and synthetic cannabinoids in pediatric populations, focusing on their pharmacology, clinical presentations, and management strategies. The narrative approach was chosen to allow for a broader integration of existing knowledge, incorporating diverse perspectives from clinical, pharmacological, and public health literature. Unlike systematic reviews, which are often constrained by rigid inclusion criteria, this method enables the exploration of emerging trends, gaps, and contextual factors in the field. By emphasizing the risks associated with these substances in children and adolescents, this review seeks to inform healthcare providers and policymakers, offering a detailed understanding that supports more effective prevention and intervention efforts.

## 2. Materials and Methods

### 2.1. Study Design

This narrative review explores the toxicological effects of synthetic cathinones and cannabinoids in pediatric populations, focusing on their neurotoxic, psychiatric, and systemic impacts. This study adopts a structured approach to identifying, evaluating, and synthesizing relevant literature (Table 1 and Table 2).

### 2.2. Search Strategy

A comprehensive literature search was conducted in the PubMed (via MEDLINE) and Scopus databases between January 2010 and September 2024. The following search string was used:

“synthetic cathinones” OR “mephedrone” OR “MDPV” OR “bath salts” AND “toxicity” AND (“pediatric” OR “adolescent”) “synthetic cannabinoids” OR “Spice” OR “K2” AND “toxicity” AND (“pediatric” OR “adolescent”). Inclusion Criteria were Peer-reviewed articles in English; studies on pediatric populations (0–18 years); articles addressing toxicological effects of synthetic cathinones or cannabinoids; case reports, case series, observational studies, and studies reporting clinical outcomes. Exclusion Criteria: Non-English articles; studies focused solely on adults; non-original research, such as commentaries and editorials; review articles; experimental studies on animals or in vitro models.

### 2.3. Screening and Selection

The database search yielded 365 articles (182 on synthetic cathinones and 183 on synthetic cannabinoids). After duplicate removal, 308 unique records were screened based on titles and abstracts. A total of 110 articles underwent full-text review, and 13 were included in this review: 5 studies on synthetic cathinones and 8 on synthetic cannabinoids.

### 2.4. Data Extraction

Two independent reviewers extracted data using a standardized form. The parameters included: study design and methodology, population characteristics (age, gender), exposure type and substance(s) involved, toxicological outcomes (e.g., seizures, psychosis, systemic effects), and clinical management and outcomes. Discrepancies were resolved through discussion with a third reviewer.

### 2.5. Analysis

The narrative synthesis method was employed to collate and interpret the findings. Studies were grouped into two domains:

- Neurotoxic and Psychiatric Effects: Studies highlighting neurological and psychological outcomes.
- Medical Effects: Studies focusing on systemic and physiological complications.

To clarify the selection of articles, we adopted a rigorous screening process to ensure the highest quality and clinical relevance of the studies included. While the initial search yielded 365 articles, only 13 met the inclusion criteria after a thorough review. This reflects both the focused nature of our review and the limited availability of pediatric-specific studies in this field. During the selection process, many studies were excluded because they focused predominantly on adult cases, and our review intentionally aimed to provide insights exclusively on the pediatric and adolescent population. The inclusion criteria prioritized studies that directly addressed the toxicological effects of synthetic cathinones and cannabinoids in pediatric populations and reported clinical outcomes. Case reports, case series, and observational studies were included to ensure comprehensive coverage of the available evidence. Review articles and non-original research were excluded to avoid redundancy, while animal and in vitro studies were not considered, as our focus was on clinical applicability to pediatric patients. The relatively small number of studies highlights a critical gap in the literature, underscoring the need for further research in this area. Nonetheless, the selected studies provide a robust foundation for the synthesis of current knowledge on this important topic.

## 3. Results

### 3.1. Clinical and Neurological Risks of Synthetic Cathinones: Acute and Delayed Manifestations 

Synthetic cathinones, often referred to as “bath salts”, are associated with significant risks, particularly for younger populations. In a study by Tekulve et al. [48], the focus was on the prevalence and severity of seizures among adolescents exposed to these substances. The findings reveal that seizures, ranging from isolated events to life-threatening status epilepticus, were frequently associated with fever and acidosis, likely linked to the sympathomimetic toxicity of synthetic cathinones. Co-ingestion of substances such as THC, alcohol, and opioids further aggravated the outcomes. The presence of fever was identified as a critical risk factor for seizures, emphasizing the need for prompt and aggressive medical intervention.

Similarly, Dargan et al. [60] explored the widespread use of mephedrone, a synthetic cathinone, among students prior to its regulation in the UK. The study found the drug’s popularity was fueled by its stimulant properties and legal status at the time, particularly among adolescents and young adults. However, adverse effects were common, including local irritations such as sore nasal passages and systemic issues like paranoia, hallucinations, and addiction. Although seizures were less frequently reported in this study, their occurrence still underscored the drug’s neurotoxic potential. Mephedrone’s accessibility, facilitated by street dealers and online sources, contributed significantly to its impact.

In a study by Wood et al. [61], acute mephedrone toxicity was analyzed in a series of cases with analytically confirmed exposure. The patients exhibited symptoms consistent with sympathomimetic toxidrome, such as hypertension, tachycardia, agitation, and, in one instance, seizures. These effects mirror those caused by other stimulant drugs, like MDMA and cocaine. The study highlights the urgent need for analytical confirmation in suspected novel psychoactive drug toxicity cases to better inform clinical and legislative decisions.

Similarly, Osterhoudt and Cook [62] presented a case of a 16-year-old boy exhibiting severe agitation, paranoia, and physical symptoms such as tachycardia and tremors after using “bath salts” containing methylenedioxypyrovalerone (MDPV). The boy’s symptoms reflected a sympathomimetic toxidrome, with evidence of prolonged neurological and psychological disturbances. The study underscores the deceptive marketing of such substances and the challenges posed by their unregulated availability, particularly among minors.

In the case report by Winerdal et al. [63], a 17-year-old girl with no prior psychiatric history developed delayed-onset catatonia following recreational use of “bath salts” and ethyl chloride. Initially presenting with confusion, hallucinations, and anxiety, her symptoms progressed within days to include speech delays, motor impairments, and classic catatonic features such as waxy flexibility and mutism. Despite initial treatment with antipsychotics, her condition deteriorated until a combination of lorazepam, memantine, and lithium yielded a full recovery over six weeks.

The pathophysiology underlying this reaction was hypothesized to involve mephedrone’s potent stimulation of serotonin, dopamine, and glutamate pathways, which may have triggered receptor adaptations leading to the delayed onset of catatonia. This case highlights not only the severe potential for neuropsychiatric harm from synthetic cathinones but also the challenges of diagnosis and treatment, as symptoms may mimic or worsen under traditional psychotropic interventions.

These findings emphasize the need for heightened awareness of the delayed and complex effects of designer drugs in vulnerable populations, as well as the potential utility of NMDA receptor modulators and mood stabilizers in managing such cases.

### 3.2. Synthetic Cannabinoids and Adverse Effects: Clinical Presentations and Risk Patterns

Synthetic cannabinoids, often marketed under names like “K2” and “Spice”, have emerged as a dangerous and unpredictable alternative to natural cannabis, particularly among adolescents. A case described by Vearrier and Osterhoudt [64] illustrates the alarming effects of these substances. A 17-year-old girl presented to the emergency department after using synthetic cannabinoids, experiencing severe agitation, tachycardia, and mild hypertension—symptoms inconsistent with typical marijuana intoxication. These effects were linked to the synthetic cannabinoid JWH-018, known for its potent affinity for CB1 receptors in the brain, which far exceeds that of THC. While her symptoms resolved with supportive care, the case highlights the profound physiological impact and unpredictability of synthetic cannabinoids, marketed deceptively as “herbal incense” or “legal highs”.

Similarly, Oluwabusi et al. [65] reported two cases of adolescent psychosis triggered by synthetic cannabinoid use. The first involved a 16-year-old boy with a family history of schizophrenia and bipolar disorder, who developed paranoia, hallucinations, and mood instability after using “K2”. Although initially stabilized with antipsychotic medication, he relapsed due to noncompliance and continued use of the drug, requiring inpatient treatment for recovery. The second case involved a 17-year-old boy with a similar family history, who experienced severe anxiety, disorganized behavior, and cognitive impairment after smoking “Spice”. He also relapsed following discontinuation of antipsychotic therapy and resumed drug use but recovered with structured inpatient care.

Jinwala and Gupta [66] reported two cases of respiratory depression linked to synthetic cannabis use. In one instance, a 19-year-old man experienced altered mental status, seizure, and severe respiratory depression requiring intubation after smoking a synthetic cannabis product marketed as “K2”. Similarly, a 15-year-old boy suffered from respiratory failure after consuming alcohol and synthetic cannabis at a party, requiring mechanical ventilation. These cases underscore the serious physiological risks posed by synthetic cannabinoids, including their impact on the central nervous system (CNS) and respiratory function.

A broader investigation by Gilley et al. [70] provides a comprehensive picture of synthetic cannabinoid exposure in adolescents. Drawing data from the Toxicology Investigators Consortium (ToxIC) database, the study documented 75 cases of synthetic cannabinoid toxicity in patients aged 12–19 years. The majority exhibited severe CNS effects, including agitation, delirium, psychosis, seizures, and coma. Notably, respiratory depression was observed in 8% of cases, with 12% requiring mechanical ventilation. The lack of a consistent toxidrome and the absence of rapid drug detection tools complicate diagnosis and treatment, leaving clinicians reliant on supportive care. The study highlights the potency of synthetic cannabinoids, which can activate CB1 receptors up to 200 times more strongly than natural cannabis, resulting in unpredictable and life-threatening effects. Dinleyici et al. [71] presented a harrowing case involving a 3-month-old infant who was exposed to Bonzai, a potent synthetic cannabinoid, through environmental contamination from parental use. The infant, although initially asymptomatic, tested positive for high levels of the drug in blood and urine, likely transferred via breast milk or passive inhalation. This case highlighted the severe risks of neglect and abuse in homes with parental drug use, underscoring the need for immediate protective interventions and legal actions to safeguard children in such environments.

Similarly, Cohen et al. [69] described three cases of adolescent intoxication due to synthetic cannabinoids. A 16-year-old girl developed catatonia and tachycardia after smoking “K2”, requiring intravenous benzodiazepine treatment. An 18-year-old boy exhibited agitation, diaphoresis, and aggression at a party after smoking “Spice”, while another adolescent suffered dystonia and confusion following synthetic cannabinoid use. All cases recovered with supportive care, but the presentations demonstrated the potent and unpredictable neuropsychiatric and systemic effects of these substances, which often mimic but exceed those of natural cannabis. Clark et al. [67] described several cases of myocardial ischemia in teenagers following K2 use. One 15-year-old presented with altered mental status, showing significant ST elevation on an electrocardiogram (ECG) and elevated troponin levels, indicative of cardiac injury. Another 16-year-old boy exhibited similar ECG changes but had normal troponin levels, while a third case involved a 17-year-old with both chest pain and significant ECG abnormalities. Although none of the patients required invasive interventions, the findings underscore the significant risk of myocardial ischemia associated with synthetic cannabinoids. These cases highlight the drug’s ability to disturb myocardial oxygen supply and demand, potentially exacerbating underlying cardiovascular vulnerabilities.

Adair et al. [68] presented two contrasting cases demonstrating the diverse spectrum of synthetic cannabinoid toxicity. The first case involved a 17-year-old male with autism and bipolar disorder, who experienced acute psychosis, agitation, and rhabdomyolysis after smoking synthetic cannabinoids. The patient required intensive care for stabilization but recovered without lasting complications. The second case involved a 12-month-old infant exposed indirectly through a parent’s use of synthetic cannabinoids. The child exhibited episodes of somnolence, apnea, and agitation, necessitating intubation and close monitoring. This case underscores the risks of passive exposure in vulnerable populations, particularly young children in environments with synthetic drug use.

## 4. Discussion

### 4.1. Neuropsychiatric and Neurological Effects

#### 4.1.1. Synthetic Cathinones

Synthetic cathinones, including mephedrone and methylenedioxypyrovalerone (MDPV), have emerged as potent stimulants with a range of acute and chronic neurotoxic effects. Studies by Tekulve et al. [59] and Wood et al. [61] emphasize their association with seizures and sympathomimetic toxicity. These outcomes are consistent with their pharmacological action as strong monoamine releasers and reuptake inhibitors, which lead to heightened dopaminergic and serotonergic activity. Such alterations can lower seizure thresholds, particularly in younger populations, where the developing brain is more vulnerable to excitotoxic insults.

The cases presented by some authors [60,61,62] extend the understanding of synthetic cathinones’ psychiatric consequences, including paranoia, agitation, and catatonia. The delayed-onset catatonia described by Winerdal et al. [63] highlights a potential mechanism involving long-term receptor dysregulation, which aligns with earlier reports of excitotoxicity and oxidative stress induced by synthetic cathinones. These findings resonate with prior studies indicating that synthetic cathinones can precipitate persistent neuropsychiatric disorders, especially in individuals with predisposing factors such as genetic vulnerabilities or concurrent substance use.

#### 4.1.2. Synthetic Cannabinoids

Synthetic cannabinoids, such as JWH-018 and those found in “K2” and “Spice”, exhibit even greater affinity for CB1 receptors than natural THC, resulting in exaggerated and often unpredictable neuropsychiatric effects. The psychosis described by Oluwabusi et al. [65] and the extreme behavioral disturbances reported by Adair et al. [68] and Cohen et al. [69] align with broader findings in the literature, which indicate a high risk of transient and persistent psychosis following synthetic cannabinoid use. These substances appear particularly dangerous for individuals with underlying vulnerabilities, such as family histories of schizophrenia or mood disorders.

Authorized cannabinoids, such as dronabinol, nabilone, and cannabidiol (CBD), are characterized by well-defined pharmacological profiles, controlled dosages, and established safety margins, as evidenced by clinical trials and regulatory approval. These compounds act primarily as partial agonists or antagonists at cannabinoid receptors (CB1 and CB2), contributing to predictable and manageable effects. For example, dronabinol and nabilone exhibit partial CB1 agonism, limiting their psychoactive and adverse effects

The catatonia observed in synthetic cannabinoid users further emphasizes the substances’ potential to dysregulate key neurochemical pathways, particularly in the endocannabinoid and dopaminergic systems. These effects may be mediated by their full agonism at CB1 receptors, contrasting with THC’s partial agonism, and are often compounded by the variable chemical composition of these products [64].

### 4.2. Medical Effects

#### 4.2.1. Synthetic Cathinones: Systemic and Sympathomimetic Toxicity

Synthetic cathinones, widely marketed as “bath salts”, exert profound stimulant effects through their action as potent monoamine releasers and reuptake inhibitors. As Dargan et al. and Wood et al. [60,61] noted, common clinical presentations include tachycardia, hypertension, and agitation, forming the hallmark of sympathomimetic toxidrome. These effects mimic those of other stimulants like MDMA and cocaine but are often more exaggerated due to the full agonist properties of cathinones at monoamine transporters.

The systemic complications described by Tekulve et al. [59] highlight the significant risks of co-ingestion, with synthetic cathinones amplifying the effects of other substances like THC and alcohol. These interactions often lead to severe outcomes such as respiratory distress and hyperthermia. Such cases underscore the challenges clinicians face in managing polysubstance toxicity, where the combined effects of multiple drugs exacerbate systemic strain, particularly on cardiovascular and respiratory systems.

#### 4.2.2. Synthetic Cannabinoids: Cardiovascular and Multisystem Impact

Synthetic cannabinoids, including compounds found in “K2” and “Spice”, display a unique profile of medical effects due to their potent agonism at CB1 and CB2 receptors. Clark et al. [67] reported cases of myocardial ischemia in adolescents, characterized by ECG abnormalities, chest pain, and elevated troponin levels. These findings suggest that synthetic cannabinoids can significantly disrupt myocardial oxygen supply and demand, increasing the risk of cardiac injury even in young, otherwise healthy individuals. This aligns with prior studies indicating that synthetic cannabinoids can exacerbate underlying cardiovascular vulnerabilities.

Respiratory complications, as documented by Jinwala and Gupta [66], are another critical concern. Cases of respiratory depression requiring mechanical ventilation highlight the potential for synthetic cannabinoids to severely impair central respiratory regulation. Such risks are heightened by co-ingestion with substances like alcohol, which can compound their depressant effects.

Adair et al. [68] expanded the understanding of synthetic cannabinoid toxicity by documenting systemic complications such as rhabdomyolysis in a 17-year-old male, indicative of severe muscle breakdown and potential renal strain. The case of apnea and somnolence in a 12-month-old infant exposed passively to synthetic cannabinoids underscores the extreme vulnerability of pediatric populations, who may experience severe outcomes even from indirect exposure [71].

### 4.3. Clinical Relevance

Synthetic cathinones and cannabinoids pose a significant and growing challenge for clinicians and public health professionals, particularly due to their unpredictable and severe effects in pediatric populations. These substances, with their potent neuropsychiatric and systemic impacts, demand tailored diagnostic and management approaches to mitigate their harm effectively. Synthetic cathinones, such as mephedrone and methylenedioxypyrovalerone (MDPV), are strong monoamine releasers and reuptake inhibitors that elevate dopaminergic and serotonergic activity. This mechanism underpins their well-documented association with seizures and sympathomimetic toxicity, especially in younger individuals whose developing brains are more vulnerable to excitotoxic insults [60,61,62]. Seizures are a prominent feature, often complicated by psychiatric symptoms such as paranoia, agitation, and catatonia. The delayed-onset catatonia described in some cases underscores the long-term neuropsychiatric risks of these substances, particularly in individuals with genetic predispositions or concurrent substance use [64]. Similarly, synthetic cannabinoids, including compounds like JWH-018, present a distinct but equally significant threat. Their greater affinity for CB1 receptors compared to natural THC often results in exaggerated neuropsychiatric effects, such as psychosis, agitation, and behavioral disturbances [66,67]. Adolescents without prior psychiatric histories are not immune, with sudden onset symptoms like catatonia being a frequent presentation. The deceptive marketing of these substances as “legal highs” further complicates their identification and management, necessitating increased awareness among healthcare providers. In addition to their neuropsychiatric effects, both synthetic cathinones and cannabinoids carry profound systemic risks. Synthetic cathinones can mimic traditional stimulants like cocaine, but their heightened sympathomimetic toxicity—characterized by tachycardia, hypertension, and hyperthermia—can lead to life-threatening complications [60,61]. Co-ingestion with substances such as THC or alcohol exacerbates these effects, creating challenges in clinical management. Synthetic cannabinoids, on the other hand, are strongly associated with cardiovascular complications like myocardial ischemia, as well as respiratory depression, requiring intensive care [68,70,71]. These risks highlight the critical importance of early recognition and timely intervention in clinical settings. One of the primary barriers to effective management is the lack of routine toxicological screening for synthetic substances. Negative results on conventional toxicology tests often mislead clinicians, delaying appropriate treatment. Moreover, the rapid evolution of synthetic drug formulations often outpaces regulatory frameworks, complicating efforts to control their production and distribution. Despite these challenges, actionable insights emerge from this review. Clinicians should maintain a high index of suspicion for synthetic substance use in pediatric patients presenting with unexplained psychiatric or systemic symptoms. This is particularly important when symptoms resemble cannabis intoxication but routine toxicology screens are negative. Early recognition and comprehensive monitoring are essential, alongside the implementation of tailored management protocols such as benzodiazepines and antipsychotics to address seizures, psychosis, and catatonia effectively. In more severe cases, supportive care and intensive monitoring may be required to manage systemic complications such as respiratory depression or cardiovascular instability. Public health efforts are equally crucial. Educational initiatives targeting adolescents, parents, and educators must emphasize the unique risks posed by synthetic cannabinoids and cathinones. Awareness campaigns should address their potential for severe neuropsychiatric and systemic effects, alongside the deceptive marketing strategies used to promote their use. Enhanced surveillance systems are needed to track usage patterns, identify emerging substances, and guide evidence-based policy responses [72,73,74,75,76]. Synthetic cannabinoids (SCPs) and cathinones present unique yet overlapping challenges. SCP use mimics cannabis intoxication but is often associated with symptoms like anxiety and mood disturbances, which are less typical of natural cannabis. Evidence suggests a link between SCP use and new-onset psychosis or the exacerbation of pre-existing conditions, although definitive causation remains to be established [77,78,79]. Pediatric patients presenting with sudden psychosis, negative toxicology screens, or signs of cannabinoid intoxication should prompt clinicians to consider SCP use as a differential diagnosis. Similarly, synthetic cathinones mimic stimulant effects but pose heightened risks of seizures, psychosis, and sympathomimetic toxidrome. Pediatric patients often experience more severe manifestations due to their developmental vulnerabilities, further emphasizing the necessity of tailored clinical approaches. The proliferation of synthetic cannabinoids and cathinones in the marketplace underscores the need for robust regulatory measures and public health interventions. Manufacturers continuously innovate to evade existing laws, producing compounds that are increasingly potent and dangerous. Their accessibility and affordability, coupled with deceptive marketing as legal alternatives, exacerbate the risks associated with these substances [72,73,74,75].

Finally, it is important to keep in mind that the development of psychopathological and neurological phenomena, as well as the mechanisms of intoxication, are strongly influenced by the frequency of use and the dosage consumed (although it is not possible to define a precise threshold dosage). Moreover, young age, male sex, and a high number of hospitalizations are significant predictive factors for the future development of psychosis [80].

## 5. Strengths and Limitations

This narrative review provides a comprehensive synthesis of the current knowledge on synthetic cathinones and cannabinoids in pediatric and adolescent populations. A key strength of this review lies in its broad scope, integrating findings from clinical, pharmacological, and public health perspectives to highlight the multifaceted challenges posed by these substances. By adopting a narrative approach, the review allows for the inclusion of diverse study designs, such as case reports, observational studies, and reviews, which provide nuanced insights into clinical presentations and management strategies that might not be captured in systematic reviews. Furthermore, the focus on pediatric populations fills a critical gap in the literature, emphasizing the unique vulnerabilities and needs of this group. However, certain limitations must be acknowledged. The narrative methodology, while flexible, does not provide the same level of rigor in evidence synthesis as systematic reviews, potentially introducing selection bias. Additionally, the reliance on published case studies and reports may limit the generalizability of findings, as these often highlight severe or atypical cases. The heterogeneity of included studies, in terms of population, exposure types, and outcomes, poses challenges for drawing definitive conclusions or establishing causal relationships. Moreover, the limited number of studies specifically focused on pediatric and adolescent populations restricts the ability to comprehensively address the scope of the issue and highlights the urgent need for more robust, large-scale research. Finally, the lack of standardized toxicological screening for synthetic substances across studies further complicates the interpretation of results and underscores the need for future research to address these gaps.

## 6. Conclusions

The findings of this review underscore the urgent need for tailored clinical and public health strategies to address the challenges posed by synthetic cathinones and cannabinoids in pediatric populations. These substances, while distinct in their pharmacological profiles, share commonalities in their unpredictable and severe effects, the gaps they expose in current medical frameworks, and the systemic responses required to mitigate their impact.

Several initiatives for intervention and prevention are already in place, though they require further expansion and integration. These include public health campaigns aimed at raising awareness about the risks of synthetic substances among adolescents, parents, and educators. School-based programs focused on substance abuse prevention, combined with early screening and intervention in at-risk youth, have shown promise. Additionally, robust regulatory measures, such as the continuous monitoring and banning of newly emerging substances, are essential to curbing their accessibility. However, significant gaps remain, particularly in the areas of long-term impact and effective therapeutic approaches. Future research should prioritize longitudinal studies to explore the chronic effects of these substances on neurodevelopment and mental health. Investigating the efficacy of tailored intervention programs for specific subgroups, such as adolescents with pre-existing mental health conditions, would be particularly beneficial. Furthermore, enhanced toxicological screening methods and real-time surveillance systems could aid in the early identification of trends in synthetic substance use, enabling more timely and targeted responses [81,82].

Finally, despite growing knowledge, substantial gaps remain in understanding the full scope of the toxicological effects of synthetic cathinones and cannabinoids, particularly in pediatric populations. Further research is essential to establish causal links between synthetic cannabinoid use and psychosis, develop effective prevention strategies targeting youth, inform treatment protocols tailored to the unique presentations of these substances in pediatric cases, and guide regulatory frameworks that can adapt to the rapid evolution of synthetic drug formulations.

## Figures and Tables

**Table 1:** Summary of studies on synthetic cathinones in pediatric and adolescent populations, highlighting study types and key findings.

Authors	Study Type	Number of Patients	Key Findings
Tekulve et al. (2014) [59]	Retrospective study based on the American Association of Poison Control Centers (AAPCC) database.	1328 adolescent (age 17 ± 2)	Seizures occurred in 5.5% of cases, with most being single episodes (50.7%), followed by multiple seizures (39.7%) and status epilepticus (9.6%). Fever and acidosis were strongly associated with seizures, and 45% of cases involved co-ingestants like THC, alcohol, or opioids.
Dargan et al. (2010) [60]	Questionnaire-based survey	349 adolescent students (14.0 years ± 1.1) and 657 college/university students (20.5 years ± 6.5 years)	20.3% had used mephedrone at least once, with 56% reporting adverse effects such as paranoia, sore nasal passages, and palpitations. 17.6% reported addiction or dependence symptoms.
Wood et al. (2010) [61]	Case series with analytical confirmation	7 adolescents 1 patients aged 16 Others aged between 17 and 36 years	Acute mephedrone toxicity linked to Agitation (4), palpitations (2), chest pain (2), seizures (1); tachycardia (5), hypertension (3). Outcomes: 4 discharged, 2 observed, 1 ICU fatality.
Osterhoudt and Cook (2011) [62]	Case report	1 adolescent (16 years-old male)	16-year-old boy exhibited agitation, paranoia, tachycardia, and tremors after 3,4-methylenedioxypyrovalerone (MDPV) use; prolonged neuropsychiatric disturbances.
Winerdal et al. (2024) [63]	Case report	1 adolescent, a 17-year-old female	Delayed-onset catatonia after exposure to ‘bath salts’; linked to serotonergic and glutamatergic dysregulation.

**Table 2:** Summary of studies on synthetic cannabinoids in pediatric and adolescent populations, highlighting study types and key findings.

Authors	Study Type	Number of Patients	Key Findings
Vearrier and Osterhoudt (2010) [64]	Case report	17-year-old female	Severe agitation, tachycardia, and hypertension after synthetic cannabinoid JWH-018 use; symptoms inconsistent with typical cannabis intoxication.
Oluwabusi et al. (2012) [65]	Two Case report	Two adolescent males aged 16 and 17	Both presented with psychotic symptoms including agitation, delusions, hallucinations, and cognitive impairment following the use of synthetic cannabinoids (K2/Spice).
Jinwala and Gupta (2012) [66]	Two Case report	Two adolescents, aged 15 and 19	Respiratory depression requiring Mechanical ventilation in adolescents after synthetic cannabinoid use, often with co-ingestion of alcohol.
Clark et al. (2015) [67]	Case series	Eight adolescent males aged 15–18	Altered mental status, chest pain, palpitations, shortness of breath, syncope, and seizures. ECG findings included ST elevations, T wave inversions, and sinus tachycardia. Troponin levels were elevated in some cases
Adair et al. (2016) [68]	Two case reports	Two pediatric cases (17-year-old male and 12-month-old male)	Psychosis and rhabdomyolysis in a 17-year-old boy; apnea and somnolence in a 12-month-old exposed indirectly through parental use.
Cohen et al. (2012) [69]	Three case reports	Three adolescents (two aged 16, one aged 18)	Catatonia, aggression, and dystonia in adolescents after synthetic cannabinoid use; resolved with supportive care.
Gilley et al. (2018) [70]	Observational study	75 adolescents aged 12–19 (66% aged 16–19; 91% male)	Predominantly neuropsychiatric, including CNS depression (29%), agitation (24%), delirium/toxic psychosis (21%), and seizures (15%). Tachycardia occurred in 16%, respiratory depression in 8%, and rhabdomyolysis in 5%
Dinleyici et al. (2018) [71]	Case report	A 3-month-old male infant.	3-month-old infant exposed to Bonzai (JWH-18) through environmental contamination; tested positive for high levels of the drug.

## Data Availability

The data presented in this study are available on request from the corresponding author. The data are not publicly available due to privacy reasons.
